# The brain dynamics of linguistic computation

**DOI:** 10.3389/fpsyg.2015.01515

**Published:** 2015-10-13

**Authors:** Elliot Murphy

**Affiliations:** Division of Psychology and Language Sciences, University College LondonLondon, UK

**Keywords:** neural oscillations, biolinguistics, syntax, dynome, theta, alpha, beta, gamma

## Abstract

Neural oscillations at distinct frequencies are increasingly being related to a number of basic and higher cognitive faculties. Oscillations enable the construction of coherently organized neuronal assemblies through establishing transitory temporal correlations. By exploring the elementary operations of the language faculty—labeling, concatenation, cyclic transfer—alongside neural dynamics, a new model of linguistic computation is proposed. It is argued that the universality of language, and the true biological source of Universal Grammar, is not to be found purely in the genome as has long been suggested, but more specifically within the extraordinarily preserved nature of mammalian brain rhythms employed in the computation of linguistic structures. Computational-representational theories are used as a guide in investigating the neurobiological foundations of the human “cognome”—the set of computations performed by the nervous system—and new directions are suggested for how the dynamics of the brain (the “dynome”) operate and execute linguistic operations. The extent to which brain rhythms are the suitable neuronal processes which can capture the computational properties of the human language faculty is considered against a backdrop of existing cartographic research into the localization of linguistic interpretation. Particular focus is placed on labeling, the operation elsewhere argued to be species-specific. A Basic Label model of the human cognome-dynome is proposed, leading to clear, causally-addressable empirical predictions, to be investigated by a suggested research program, Dynamic Cognomics. In addition, a distinction between minimal and maximal degrees of explanation is introduced to differentiate between the depth of analysis provided by cartographic, rhythmic, neurochemical, and other approaches to computation.

The argument for placing language at the center of investigations into human cognition has by now been pushed on a number of fronts, from palaeoanthropology to philosophy (McGilvray, 2013; Hauser et al., 2014). In contrast, attempts to place the brain at the center of the language sciences have been met with suspicion and even ridicule, typically due to the observation that higher cognitive constructs like *verb* and *phrase* cannot presently be made commensurable with lower-level neurophysiological structures like *dendrite* and *cortical column*. Substantial engagement with the biology literature is a feature still lacking in departments of linguistics, despite the Minimalist Program's narrowing of the boundaries between the computational and conceptual capacities of humans and non-humans (Chomsky, 1995, 2012, 2015b).

One of the core motivations linguists have for leaving aside biology and keeping to computational investigations arises from Poeppel (2012) and Chomsky's (2000) insightful discussions concerning philosophy of science, theoretical reduction, and unification. These authors point out that, as with the reduction of physics to an unaltered chemistry in the early years of the twentieth century, it may well be that a new neurobiology yielded by a “Galilean” revolution is required for commensurability with the computational theories of syntacticians to be achieved, rather than a revolutionized theory of language. But the common claim that linguistics is biology at a suitable level of abstraction (Berwick, 2011) is also used to effectively get linguists “off the hook” of directly exploring the biology of language, satisfied as many are with concluding that this is purely the job of neuroscience. Yet if neuroscientists are not guided by the concerns of computationalists across the cognitive sciences, and not just linguistics, then there is little reason to believe that this goal will ever be achieved. As Lenneberg (1964, p. 76) noted, “[n]othing is gained by labeling the propensity for language as biological unless we can use this insight for new research directions—unless more specific correlates can be uncovered.”

## Dynamic cognomics: preliminary remarks

The central argument of this paper will be that recent developments in brain dynamics and neurochemistry can provide the type of framework needed to meet Poeppel and Embick's (2005) challenge of “granularity” mismatch, or the problem of reconciling the primitives of neuroscience with the primitives of linguistics (see also Fitch, 2009; Poeppel, 2011). The brain simply does not know what *syntax* or *phonology* are, and these concepts are much too coarse to be implemented neurally. In 1996, Poeppel noted of cell assemblies and oscillations that “it is unclear whether these are the right biological categories to account for cognition” (1996, p. 643), but by now the oscillation literature has sufficiently expanded to incorporate numerous cognitive processes.

Linguistics can direct the brain sciences insofar as its insights into the universality of operations like concatenation (set-formation) inform the goals of neurobiology, while the brain sciences can direct linguistics insofar as they place constraints on what possible operations neuronal assemblies and their oscillations can perform. While linguists should focus on making their claims about language biologically feasible, neuroscientists should conversely ensure they do not sideline the notion of computation, as stressed by Gallistel and King (2009).

In order to explore these manifold agendas, I will adopt the multidisciplinary approach promoted by Boeckx and Theofanopoulou (2014), which endorses an interweaving of the sciences concerned with the following topics: the computations performed by the human nervous system (the “cognome”; Poeppel, 2012), brain dynamics (the “dynome”; Kopell et al., 2014), neural wiring (the “connectome”; Seung, 2012) and genomics. This framework exposes the misleading nature of common questions surrounding whether the brain's wiring “makes us who we are,” which have been given an impetus by calls from Seung (2012) and others for a map of the connectome. The connectome constrains the *kinds* of operations performed by the nervous system, but it cannot reveal *what* operations in particular are performed. What is needed, as Seung himself has explained, is not just a comprehensive model of neural wiring, but also neural computation, which is what a theory of the cognome can contribute (see Reimann et al., 2015 for a proposed algorithm to predict the connectome of neural microcircuits).

Bridging the two domains, I will argue, is the dynome; or what physicists would term the mesoscale, and not the microscale. The dynome is the level of brain dynamics, encompassing electrophysiology, and neural oscillations. It explores “not only *what* is connected, but *how* and in what directions regions of the brain are connected” (Kopell et al., 2014, p. 1319). The cartographic literature (e.g., fMRI and DTI studies) typically displays theoretical and empirical satisfaction with discussions of neural “activation,” “firing,” and “pathways,” keeping at a connectomic level of spatiotemporal brain nodes and edges (Bressler and Menon, 2010). The dynome adds to such a “functional connectome” an understanding of the regions involved in producing and processing brain signals. Although I will focus on brain rhythms, it should be noted that the dynome extends beyond neural oscillations and includes other temporal structures (Larson-Prior et al., 2013).

I would also like to propose that the universality of language, and the true biological source of Universal Grammar, is not to be found purely in the genome as has long been suggested (where there are surprising layers of variation; Benítez-Burraco and Boeckx, 2014a,b), but more specifically within the extraordinarily preserved nature of mammalian brain rhythms (the oscillations of mice and rats have the same pharmacological profiles as humans) likely arising from the deployment of long-diameter axons of long-range neurons (Buzsáki et al., 2013, see also Calabrese and Woolley, 2015). Such cortical and sub-cortical structures are “among the most sophisticated scalable architectures in nature” (Buzsáki et al., 2013, p. 751), with scalability referring to the ability to perform the same operations with increasing efficiency despite escalating organizational complexity. Brain rhythms, yielded in part by such structures, would therefore be expected to be capable of complex forms of information-transmission and integration.

A central question posed by this paper, then, is “Why claim that neuroscience requires a Galilean revolution in order for it to be made commensurable with linguistics when the properties of syntax may be able to be translated into rhythmic brain processes?” The current paper will suggest a new research program, Dynamic Cognomics, to explore the neurobiology of language in a deeper and more electrophysiologically explicit fashion than many existing cartographic neuroimaging studies, but some important background is needed before any concrete research goals can be drawn up.

## Cartographic directions

In Murphy (2015a) it was claimed that the ability to label linguistic structures with a categorical identity (e.g., determiner, verb, and adjective), having concatenated two elements into an unordered set, and transfer them in a cyclic fashion to the conceptual-intentional (CI) interface is the defining property of the human computational system. This perspective will be maintained here. It will be argued that modifications in oscillatory couplings and the cell assemblies targeted by such dynomic operations are a viable candidate for what brought about what could be regarded as a phase transition from single-instance set-formation (of the kind seen in birdsong) to unbounded set-formation. For instance, the phase/non-phase rhythm of syntactic computation ([C/T[*v*/V[D/N]]]), emphasized by Richards (2011), Uriagereka (2012) and Boeckx (2013), may translate well into the rhythmic processes of neural oscillations.

Since the origins of modern cognitive neuroscience, linguistic processes have been claimed to elicit numerous event-related potentials (ERPs) by psycholinguists using magnetoencephalography (MEG) and electroencephalography (EEG) (see Swaab et al., 2012 for a review). As time-frequency analysis and its Fourier transforms developed into a mainstay of “ERPology” (Luck, 2014) in the 1990s and 2000s, it became possible to test the involvement of distinct brain regions and the concomitant electrical activity for various linguistic processes, given the standard assumption that language is a cognitive system. The ERP community has spent a great deal of time decomposing the major components, such as the P600 and N400. It is taken for granted that the level of analysis provided by these “large” components does not suffice at the electrophysiological level to describe generic linguistic sub-operations. The urge to seek a finer level of granularity, then, is clearly manifested in the ERP community through EEG and MEG investigations (Lau et al., 2008), but this objective is not found in the vast majority of cartographic neuroimaging research.

In recent decades, neuroanatomical inquiry into the structures responsible for syntactic processing has led to a number of revelations concerning the biology of language. Petersson et al. (2012) reveal the inadequacy of the classical Broca-Wernicke-Lichtheim language model of the brain by noting how the language network extends to substantial parts of superior and middle temporal cortex, inferior parietal cortex, along with subcortical areas such as the basal ganglia (Balari and Lorenzo, 2013), the hippocampus and the thalamus (Theofanopoulou and Boeckx, Forthcoming a). The network is also implicated in more general cognitive systems like the default-mode network and the multiple demand system.

Brodmann area 44 and the posterior superior temporal cortex appear to be involved in a pathway which supports core syntactic computations (Friederici et al., 2006, see also Tettamanti and Weniger, 2006; Santi and Grodzinsky, 2010), with the combinatorial network being identified by Poeppel (2014) as the anterior medial temporal gyrus and anterior inferior temporal sulcus. Lieberman's (2006) “Basal Ganglia Grammar” model proposes the existence of a pattern generator whose excitation/inhibition mechanism is located in the basal ganglia. This interfaces with working memory space located in Broca's area (Santi et al., 2015). Lieberman estimates that the dorsolateral prefrontal circuit is involved in sentence comprehension, projecting from the prefrontal cortex toward the lateral dorso-medial region of the globus pallidus, and the thalamus, which projects back to the prefrontal cortex. Balari and Lorenzo (2013, pp. 100–102) have suggested that this may be the circuit used as language's computational system operating within a structure of working memory networks (Balari et al., 2012).

## Evo-devo directions

As the theory of evolution expands beyond the Modern Synthesis and into areas such as evolutionary-developmental (evo-devo) biology (Carroll, 2006; Bolker, 2008) there is in turn more potential for space for linguists to find their place within biology. In the evo-devo program, following the lead of traditional formalists such as Vicq-D'Azyr, Goethe and Owen (Amundson, 1998, 2006), natural selection is “a constantly operating background condition, but the specificity of its phenotypic outcome is provided by the developmental systems” (Pigliucci and Müller, 2010, p. 13). Evo-devo departs from Neo-Darwinian adaptationism (NDA), or “phylogenetic empiricism” (Chomsky, 1968), in that it takes the saltationist view that species are the result of punctuated genetic changes. The functionalism of NDA should also be rejected, since functions do not typically pre-exist organic form (Müller, 2008), which is determined by morphogenetic parameters such as the viscoelastic properties of cellular matrices and the kinetic activity of cellular diffusion (what Alberch termed “morphological evolution”), and which at best have what Balari and Lorenzo call a “functional potential” (2013, p. 37). Contrary to ideas in Dawkins (2006, p. 202) and Lieberman (2015), laws governing the conservation of developmental pathways should be “acknowledged with a creative character similar—if not superior—to that of natural selection” (Balari and Lorenzo, 2013, p. 115). Form often precedes function, then, and natural selection acts as a “filtering condition on pre-existent variants”; thus “arrival of the fittest, instead of survival of the fittest, is the core issue in any evolutionary study” (Narita and Fujita, 2010, P. 364, see also Bertossa, 2011).

In this connection, Rakic and Kornack (2001) observe that the phase of asymmetric cell division yielding neuronal cells differs in timing between humans and monkeys to the extent that human neuronal populations are thought to be between 8 and 16 times larger than those of monkeys. Human-specific neuronal traits include the protein ApoE4, providing stronger synaptic connections (Bufill and Carbonell, 2004). Parker and McKinney (1999) detail how the myelinisation of the neocortex occurs in humans until the age of 12, but lasts only 3.5 years in rhesus monkeys. Zhang et al. (2011) also propose the existence of 1241 primate-specific genes, 280 of which are human-specific. 54% of these human-specific genes are upregulated in a brain area implicated in higher cognition, the prefrontal cortex. These new genes are “much more likely to be involved in gene regulation” (Diller and Cann, 2013, p. 256), a major topic in evo-devo.

Recent research in avian genomics suggests that the evolution of externalization may also not be as difficult as typically considered by generative grammarians. Pfenning et al. (2014, p. 1333) demonstrated that the profiles of transcription genes in vocal learners can be aligned, with 50 genes being shared between humans and birds which are “enriched in motor control and neural connectivity functions.” Both humans and birds appear to have converged on identical solutions to vocal learning; a remarkable finding considering the 310 million year gap separating birds from humans. In summary, a slight epigenetic change, termed the “Small Bang” in Murphy (2015a), could have produced an alteration in the human computational system. The next section will consider how these operations could be implemented in the brain.

## Rhythmic directions

How much physiological detail is required to capture the operations of the language faculty? Theofanopoulou and Boeckx (Forthcoming b) claim that studying neural dynamics only at the level of brain waves is sufficient, but as demonstrated below, a more refined biophysical picture is not only possible but in fact necessary to adequately explain the origins of linguistic computations like concatenation, cyclic transfer and labeling. What is needed is not just a neuroscience of language, but a *neurophysiology* of language. For instance, at the most general mesoscopic physiological level of local neuronal groups, synchronized firing patterns result in coordinated input into other cortical areas, which gives rise to the large-amplitude oscillations of the local field potential. Inhibitory interneurons play an important role in producing neural ensemble synchrony by generating a narrow window for effective excitation and rhythmically modulating the firing rate of excitatory neurons. Interneurons place constraints on the oscillations responsible, as argued here, for computation. Subthreshold membrane potential resonance may also contribute to oscillatory activity by facilitating synchronous activity of neighboring neurons. As Cannon et al. (2014, p. 705) note, “the physiology underlying brain rhythms plays an essential role in how these rhythms facilitate some cognitive operations.”

Shifting focus from neuroimaging to more recent investigations of brain oscillations may provide a welcome (but as yet tenuous) way of reconstructing in neural terms the operations of theoretical linguistics. Brain rhythms “have come of age,” as Buzsáki and Freeman (2015, p. v) put it. They reflect synchronized fluctuations in neuronal excitability and are grouped by frequency, with the most common rhythms being delta (δ: ~0.5–4 Hz), theta (θ: ~4–10 Hz), alpha (α: ~8–12 Hz), beta (β: ~10–30 Hz), and gamma (γ: ~30–100 Hz). These are generated by various cortical and subcortical structures, and form a hierarchical structure since slow rhythms phase-modulate the power of faster rhythms.

It is by now well established that neural oscillations are related to a number of basic and higher cognitive functions, for example speech perception (Giraud and Poeppel, 2012; Kayser et al., 2014). According to Giraud and Poeppel's temporal linking hypothesis, oscillation-based decoding segments information into “units of the appropriate temporal granularity” (2012, p. 511). Oscillations may consequently explain how the brain decodes continuous speech, however Giraud and Poeppel's form of dynomic research crucially centers on the segregation of phonological, and not semantic or syntactic units, which may implicate different brain areas and rhythms. The γ, θ, and δ rhythms respectively correspond closely to (sub)phonemic, syllabic and phrasal processing, as Giraud and Poeppel note, restricting their experimental inquiry to the γ and θ bands. In addition, the neural dynamics responsible for syntactic operations may be obscured by the processing of external sensory events like speech, and so different experimental designs may be required to control for this.

Oscillations have also been linked to the timing of cortical information processing (Klimesch et al., 2007). As Vaas notes, “Intrinsic oscillatory electrical activities, resonance and coherence are at the root of cognition” (2001, p. 86), with the condensing and dissolving of oscillatory bursts possibly explaining the “cinematic” nature of subjective experience (Freeman, 2015). As Poeppel has put it, the brain essentially “breathes” through oscillations. If such generic neural operations are also shown to be responsible for syntactic computations, and not just linguistic perception, this would lend weight to Hagoort's (2014) interpretation of the cartographic literature, which holds that the establishment of an axis of language production and comprehension is not justifiable. Expanding on Giraud and Poeppel's (2012, p. 511) goal of establishing a “principled relation between the time scales present in speech and the time constants underlying neuronal cortical oscillations,” one of the central challenges will be to draw up relations between oscillatory time constants and the time scales of syntactic computation. This latter topic has yet to be explored in any serious detail, possibly due to a widespread prejudice that neurolinguistic investigations of syntax must analyse phrasal units, such as noun and verb phrases, rather than the underlying operations which construct them, such as set-formation and labeling (although see Ohta et al., 2013 for an innovative approach to localizing Merge and Search operations).

### Oscillations as functional units

Recent debates about the origins of ERP component generation have led some (Tass, 2000; Makeig et al., 2002) to propose that components do not arise purely from latency-fixed polarity responses which are additive to continuing EEG responses, but rather arise through a superposition of oscillations which reset their phases in reaction to sensory input (although see Sauseng et al., 2007 for the methodological limitations of particular phase resetting claims). For our purposes, it is worth noting that this phase reset model was the first to propose a strong dependency between components and oscillations, introducing to brain dynamics a *functional* and not purely *electrophysiological* role. This immediately granted researchers the ability to transfer understanding of components (which are in turn linked to cognitive faculties) to brain rhythms whilst correspondingly inferring the nature of components from an emerging understanding of oscillations. While cognitive electrophysiologists have embraced this integrally reciprocal perspective (Klimesch et al., 2004), linguists generally remain hostile to the claim that the nature of mental computations—like components—could be explored explicitly through biophysics.

While the cognome resides at the Marrian computational level (Marr, 1982), I would like to suggest that there is in fact no algorithmic level at syntax. At most there are algorithms at the interfaces. Psycholinguistic theories can algorithmically model language processing, as Neeleman (2013) discusses, but syntax itself (being composed of operations like Concatenate, Label, and Transfer) has no need for this. Nevertheless, the dynome, with its operations of information segregation and spike timing organization, can in some sense be seen as an algorithmic level, implemented by the cellular structures of the connectome. These Marrian concerns become more vivid when we consider with Martins and Boeckx (2014) that syllables, which are unique to humans, evolved from primate lip-smacking. In terms of brain rhythms, they are both identical, yet one is human-specific and another is not. The implications for the study of labeling, not acknowledged in Murphy (2015a), are clear: only comparative investigations of domain-general neurophysiological mechanisms, and the context in which they operate, will lead to enhanced understanding of human-specific computations. There are two central approaches to the cognome-dynome one could adopt: re-construct the cognome from the bottom-up, or import linguistic constructs into a model of the dynome. I will be primarily concerned with the latter methodology, though the material reviewed and the model outlined open up the possibilities of using neurophysiology to guide linguistic investigations.

### The basic label model of the cognome-dynome

At the most general level of analysis, neural oscillations emerge from the tension between the brain's two most central principles: segregation of function and dynamic integration (de Pasquale et al., 2012). Human brains are highly complex dynamical systems with principles of cellular and electrochemical organization which range across a hierarchy of scales. The brain cannot function purely through anatomical connections—the *locus classicus* of standard neuroimaging studies—but additionally requires dynamic functional connectivity, achieved through oscillatory synchronization. Frequency bands alone are not sufficient for computation; rather, it is their interactions which are significant. Intuitive prejudices against studying complex systems in these dynamical terms abound: for instance, chemical dynamics are typically thought about in terms of reaction kinetics, being stipulated as pre-formed stable variables, ignoring the molecular composition/decomposition process.

A core feature of the brain's functional complexity is created by rhythms generated in different cortical and subcortical tissue. Oscillations denote distinct states of brain activity, while oscillatory activity reflects a dynamic interplay between the dissimilar cell types of discrete circuits (Buzsáki, 2006). Brain rhythms, with their inter-wave hierarchies, provide “a syntactical structure for the spike traffic within and across circuits at multiple time scales” (Buzsáki and Freeman, 2015, p. viii). “Phase synchronization” will additionally be a central notion to the present discussion, referring to a consistent phase coupling between two neuronal signals oscillating at a given frequency. γ band synchronization (GBS) in particular has been intensively studied due to its apparent role in phase coding and perceptual integration (Fries, 2009), and is thought to be a major process subserving a fundamental operation of cortical computation implicated in various cognitive functions. Which functions are involved depends ultimately on what neural circuits GBS operates on. The following sub-sections will present a way of exploring the operations of the cognome in terms of these dynomic operations, leading to a form of what I will call Dynamic Cognomics.

#### Concatenation

The central proposal of the model pursued here is that the interaction of brain rhythms yields linguistic computation. Lower frequencies such as the α range are known to synchronize distant cortical regions; procedures which may represent the substrates of linguistic cross-modular transactions (Kinzler and Spelke, 2007). More precisely, I will assume that the α band embeds γ rhythms generated cross-cortically, yielding a form of inter-modular conceptual combination, the electrophysiological equivalent of concatenation. The assemblies implicated by the γ range may have been influenced by the extended neocortical myelinisation discussed above, with direct effects on the network of information stored across such regions. This is consistent with recent claims that α is responsible for the binding of visuo-spatial features (Roux and Uhlhaas, 2014) and is deployed in the service of determining successful lexical decisions (Strauss et al., 2015). I will further assume that the items concatenated are also initially “lexicalized” by α-embedded cell assemblies oscillating at the γ range within supragranular layers of the default-mode network (Raichle et al., 2001).

#### Transfer

Linguists take concatenation to occur cyclically (Chomsky, 2008), and so I will additionally assume that this Spell-Out/Transfer process is realized through embedding the above γ rhythms inside the θ band, which finds its source in the hippocampus. I will adopt the claim of Theofanopoulou and Boeckx (Forthcoming b) that γ must be decoupled from the α band through the activity of the thalamic reticular nucleus for γ-θ embedding to take place. Both types of Transfer operations—Spell-Out to the sensorimotor interface, SM, Interpret to the conceptual-intentional interface, CI—will be subsumed under this approach, which at a minimum involves this desynchronization of α-generated structures and consequent θ-synchronization. Though the thalamic reticular nucleus is here identified as a core component of desynchronization, other regions may also be involved. Due to its role in γ-θ embedding in auditory processing (Nosarti et al., 2004), the posterior corpus callosum is also likely to be heavily involved in Transfer operations.

#### Labeling

Along with concatenation and transfer, there is also labeling. Two major observations have been made about this operation: (i) It is unique to humans (Murphy, 2015a); (ii) It is based on principles of minimal computation (Chomsky, 2015a). Labeling is also monotonic in that once a set has been labeled (as a verb or determiner phrase, for instance) its identity is sustained when embedded inside another set. Since labeling must take place at the point of transfer to the interfaces (to prevent a structure being a Verb Phrase at CI but a different phrase at SM), labeling must be seen as a core syntactic operation (Murphy, 2015b; Piattelli-Palmarini and Vitiello, 2015), and not emerging epiphenomenally at the interfaces, despite it having a less central role than unconstrained “Merge” (concatenation) which operates independently from either CI or SM.

As Boeckx and Theofanopoulou (2015) note, labeling was not formulated at a fine enough level in Murphy (2015a) to avoid the granularity mismatch problem. In order to correct for this, I will define labeling as the attribution to a concatenated set some categorical specification created from the Labeling Assembly, which is composed of aspects of (i) general cognitive constraints, (ii) the CI system, (iii) the cognome and (iv) the precursor lexicon (_*p*_LEX). The final of these four constituents is taken to be the set of flat and atomic “root” structures (Boeckx, 2014a), from which morphology constructs internally hierarchical words (Nóbrega and Miyagawa, 2015). When *John* is concatenated with *ran*, the labeling algorithm produces a Verb Phrase, not a Noun Phrase (see Adger, 2013; Narita, 2014a and Murphy, 2015a for further algorithmic details). This covers the basic outline of labeling, but in order to achieve a finer level of granularity it will be necessary to descend to the dynomic level, and ultimately (in the final section) the cellular level.

In dynomic terms, I will take labeling to be the slowing down of γ to β followed by β-α coupling, involving a basal ganglia-thalamic-cortical loop (see Cannon et al., 2014 for the rhythmogenesis of β in the basal ganglia). This would disinhibit the thalamic medio-dorsal nucleus via the β band. This frequency coupling arises from a relationship between oscillations which form a hierarchy such that the speed of the slower rhythm controls the power of the faster rhythm. Due to its involvement in phrasal processing, I will assume that the δ band may be involved in the later stages of this process. The role of the thalamo-cortical network as a slow rhythm generator, and hence a single dynamic and functional unit of brain oscillations, has been recently supported by Crunelli et al.'s (2015) review of the EEG literature. Accumulating evidence suggests that β holds objects, whereas γ merely generates them (Martin and Ravel, 2014). Dean et al. (2012) also show how β is an excellent candidate for comparing old and new information from distinct modalities due to its wider temporal windows; that is, it would compare phase heads (old information) with late-merged non-phasal elements like complements (new information), likely drawing on different conceptual representations and hence different “core knowledge systems” and brain regions (Spelke, 2010). Related both to Balari and Lorenzo's (2013) claim that the basal ganglia is the center of their “Central Computational Complex” and Jouen et al.'s (2013) findings that this structure is implicated in acquiring the serial response order of a sequence, Theofanopoulou and Boeckx (Forthcoming b) propose that this region holds one of the γ-supported items before slowing it down to the β frequency as a consequence of the conduction delays resulting from the surrounding neural regions. Thus the β band accomplishes the role of labels, a claim supported by findings that β activity maintains existing cognitive states (Engel and Fries, 2010). More broadly, the basal ganglia and the striatum are implicated in sequencing and chunking, with striatal structures operating at the β range (Leventhal et al., 2012). The core position occupied by the basal ganglia in this labeling model also fits well with imaging studies which have revealed the region's involvement in “syntactic complexity,” specifically the processing of type-identity intervention of matching labels, being activated in a recent fMRI study when a noun phrase similar to the dependency head in a long-distance dependency intervenes in the dependency (Santi et al., 2015). Basal ganglia nuclei in humans are also around twice as large as would be predicted for a primate of our size (Schoenemann, 2012), and since humans do not appear to have substantially more sophisticated movements than apes, this increase may well have supported higher cognitive capacities like labeling.

#### Formal considerations

Introducing new formalisms will permit a clearer explication of dynamic cognomics. Although they appear similar, what follows will have no direct bearing on, and should not be considered an extension of, standard set-theoretic notational conventions relating to such things as functional application.

First, we can notate γ-θ embedding as {θ(γ)}, with γ being embedded inside θ rhythms. If it is known how many γ cycles are to be embedded (for instance, 7), this can be notated as {θ(γ7)}. We can notate the decoupling process required to transfer concatenated structures as γ(•)α, where γ is decoupled from the α band. Frequency coupling can correspondingly be notated as γ•α. The decreasing of γ to β can be represented as γ < → β, where “ → ” refers to a state change. Post-phrasal syntactic reanalysis and wrap-up effects can be represented with ψ. Finally, the (hypothetical) cell assemblies responsible for particular lexical features, such as the [+singular] feature of *man*, can be represented as ζ[*man*(+singular)]. If it is known in which regions (cytoarchitechtonic or otherwise) such assemblies are located, this can be represented as, for instance, ζ:BA44[*man*(+singular)], while the rhythm band can be additionally represented as ζ[*man*]:γ.

We are now in a position to write a simple derivation. Take the sentence *The man is called John*. This can be represented in familiar syntactic terms as a Tense Phrase, ignoring superfluous details (e.g., morphological operations): [_*TP*_[_*DP*_ *The man*][_*T*_[_*T*_ *is*][_*VP*_ *called John*]]]. In the interests of clarity, I will put aside precise categorical concerns and denote labeled phrases with “L,” with multi-phrasal labels being italicized. Even though sentences are parsed in a left-right fashion, generative linguistics holds that syntactic derivations proceed right-left. In order to deal with this perennial psycholinguistic problem, I suggest that structures are concatenated, labeled and transferred as and when they are heard, read or otherwise perceived, and after every lexical unit a “look back” procedure is triggered to reanalyse the labels and features of each structure, denoted here by ψ (see Chesi, 2015 for a comprehensive left-right derivational proposal). In psycholinguistic terms, this may account for certain wrap-up effects which occur when subjects reach the final word of a sentence during online processing (Field, 2004). This approach is also consistent with the “one-system” contention of Lewis and Phillips (2015) that grammatical theories and language processing models describe the same cognitive system, as evidenced by the fact that grammar-parser misalignments only seem to occur as a consequence of limitations in domain-general systems such as memory access and control mechanisms. It follows that “online and offline representations are the product of a single structure-building system (the grammar) that is embedded in a general cognitive architecture, and misalignments between online (“fast”) and offline (“slow”) responses reflect the ways in which linguistic computations can fail to reflect the ideal performance of that system” (Lewis and Phillips, 2015, p. 39). This one-system hypothesis also proposes that the grammar goes through a series of structure destruction and rebuilding operations as new words are encountered; a process which aligns well with the rhythmicity of the present model and the effects of ψ.

The derivation will proceed as follows. *The* is generated by distributed γ activity in the supragranular cell assemblies responsible for its long-term storage, ζ[*the*]. This rhythm would be embedded within α activity before being transferred to the interfaces through being decoupled from α and newly embedded within hippocampal θ activity. ζ[*man*], operating at the γ range, would then be embedded within α before being transferred. The two representations would then be labeled a Determiner Phrase at the Labeling Assembly, which I will identify as the circuits connecting the thalamus, basal ganglia, prefrontal cortex, and anterior temporal regions. To achieve labeling, the embedded cycles would be slowed to the β range (γ < → β) before being coupled to β (β•α). The labeled phrase [*the man*] would be maintained in memory via the β rhythm. The subsequent material [*is called John*] would then be added in a similar fashion:

$$\begin{array}{l} \zeta [the]:\gamma \to \{\alpha (\zeta [the]:\gamma )\}\to \alpha (•)\zeta [the]:\gamma \to \{\theta (\zeta [the]:\gamma )\}\\ \zeta [man]:\gamma \to \{\alpha (\zeta [man]:\gamma )\}\to \alpha (•)\zeta [man]:\gamma \\ \to \;\{\theta (\zeta [man]:\gamma )\}\\ \left\{\theta (\zeta [the]:\gamma )(\zeta [man]:\gamma )\right\}\\ \psi \\ \gamma <\to \beta \\ \alpha •((\zeta [the]:\beta )(\zeta [man]:\beta ))\to \alpha •(\zeta {[}_{\text{L}}the\;man]:\beta )\\ \zeta [is]:\gamma \to \{\alpha (\zeta [is]:\gamma )\}\to \alpha (•)\zeta [is]:\gamma \to \{\theta (\zeta [is]:\gamma )\}\\ \gamma <\to \beta \\ \alpha •((\zeta {[}_{\text{L}}the\;man]:\beta )(\zeta [is]:\beta ))\to \alpha •(\zeta {[}_{L}{[}_{\text{L}}the\;man]{[}_{\text{L}}is]]:\beta )\\ \psi \\ \zeta [called]:\gamma \to \{\alpha (\zeta [called]:\gamma )\}\to \alpha (•)\zeta [called]:\gamma \\ \to \{\theta (\zeta [called]:\gamma )\}\\ \gamma <\to \beta \\ \alpha •((\zeta {[}_{L}{[}_{\text{L}}the\;man]{[}_{\text{L}}is]]:\beta )(\zeta [called]:\gamma ))\\ \to \alpha •(\zeta )\;{[}_{L}{[}_{\text{L}}the\;man]{[}_{L}{[}_{\text{L}}is]{[}_{\text{L}}called]]]:\beta )\\ \psi \\ \zeta [john]:\gamma \to \{\alpha (\zeta [john]:\gamma )\}\to \alpha (•)\zeta [john]:\gamma \\ \to \{\theta (\zeta [john]:\gamma )\}\\ \gamma <\to \beta \\ \alpha •((\zeta {[}_{L}{[}_{\text{L}}the\;man]{[}_{L}{[}_{\text{L}}is]{[}_{\text{L}}called]]]:\beta )(\zeta [john]:\gamma ))\\ \to \alpha •(\zeta {[}_{L}{[}_{\text{L}}the\;man]{[}_{L}{[}_{\text{L}}is]{[}_{\text{L}}called\;john]]]:\beta )\\ \psi \end{array}$$

Notice that, as with Computational Ethology (Murphy, 2015a) and recent syntactic proposals (Hornstein, 2009; Adger, 2013), labeling is here placed at the center of the dynome's linguistic operations. As a result, call the above cognome-dynome hypothesis the Basic Label model. What remains to be added to the derivation by empirical investigation are the factors of time-frequency domain and the anatomical regions of cellular assemblies (e.g., “embed γ of region *r* within α of region *s* for time *t*”). All elements in the derivation, then, are created as simple γ assemblies, and only some (namely, labeled phase heads) become more complex β assemblies; consider the difference between adverbs like *nearly* and verbs like *ran*. The rhythmic division of complexity which follows from this is supported by Honkanen et al. (2014), who demonstrated that simple objects represented in visual working memory employ the γ band, while more complex objects are represented by the β band. This oscillatory procedure also matches the generative view that phase heads have a longer derivational life than non-phase heads (Boeckx, 2014a; Narita, 2014a). The role attributed here to γ assemblies additionally finds some support in Bastiaansen and Hagoort's (2015) EEG study of semantic unification, which detected larger γ-band power for semantically coherent than semantically incongruent sentences. Larger β-band power was also found for syntactically correct sentences relative to ungrammatical sentences, lending support to the hypothesized labeling power assigned to β in the present model.

### Some empirical consequences of dynamic cognomics

Among many other forms of imaging and behavioral data, neuroimaging studies should be used as a guide for dynamic cognomic investigations. With respect to linguistic computation, the left anterior temporal lobe has been implicated in basic combinatorics (concatenation) and phrasal construction (labeling) (Bemis and Pylkkänen, 2013; Westerlund and Pylkkänen, 2014), while the posterior middle temporal gyrus is involved in lexical access (lexicalization) and ambiguity resolution (Turken and Dronkers, 2011). Given the present rhythmic perspective on linguistic computation, the much-discussed fronto-temporal language network would consequently be purely an *output* system of the above operations, not a core syntax region. Friederici (2012) holds that distinct regions of the left inferior frontal gyrus are responsible for “different” types of syntax, arguing, for instance, that the dorsal stream is only implicated in embedded structures or structures deviating from normal ordering. Yet, as the above model makes clear, the basic combinatorics are universal across syntactic structures, whether simple or complex; set-formation is still set-formation whether it is found in a small clause or a Shakespearean sonnet.

While relatively little is known about how oscillations relate to cognitive operations, significant advances could come from direct empirical investigations teasing apart γ and β from other rhythms, demonstrating a correlation with a syntactic manipulation (and perhaps a dissociation with another operation which could be linked to slower rhythms and working memory or attention processes; see Lakatos et al., 2008 for the role of oscillations in attention). Due to its high temporal and spatial resolution and signal-to-noise ratio, electrocorticography is also highly applicable to testing the Basic Label model, having been used to investigate speech production (Bouchard and Chang, 2014), language comprehension (Cervenka et al., 2011), and having been flexibly deployed both in humans and animals. In addition to the cartographic studies above, paradigms such as that in Ohta et al. (2013), which differentiate the neural correlates of concatenation and search/agreement operations, could be employed. Despite having noted the limitations of cartographic studies, an area of ongoing neurolinguistic research is the spatial scales of brain rhythms. It could be explored, for instance, whether ongoing oscillations and generic computations share the same neuronal generators. Emerging technologies to experimentally test and refine the Basic Label model include high-density electrode recordings and optogenetic tools (Chow et al., 2010; Viventi et al., 2011), along with the more traditional EEG and MEG devices. Bemis and Pylkkänen (2013) showed that between 200 and 300 ms after the presentation of a word which can be combined with a previous item, the left anterior temporal lobe is activated, implicating this region in semantic composition. This would consequently be a good estimate of when oscillation studies might detect labeling effects to arise, given the role of labels in semantic composition (Hornstein and Pietroski, 2009; Murphy, 2015b). At the most general level of lexical comprehension, EEG and MEG studies would also predictably find coherent oscillatory activation of large neuronal assemblies when processing words relative to processing pseudowords, as Pulvermüller et al. (1994) found. A level of cortical entrainment would also be predicted for non-syllabic, phrasal, and sentential structures during the auditory presentation of simple stimuli; structures which are not part of any speech stream but are rather internally constructed by the comprehender, and whose rhythmic generators would likely align closely with the regions implicated in the Basic Label model.

Neural potentials have typically been analyzed through frequency, time-frequency, and wavelet representations (Kaiser, 2010). Independent component analysis (ICA) has also been used successfully in estimating the sources of neural systems given multiple recording locations, since these systems generate independent and continuous activity and combine linearly and instantaneously (Hyvärinen and Oja, 2000). However, spatial ICA does not allow the interpretation of time-varying patterns, and in the case of EEG it also does not produce a model of “phasic events” of rhythmic activity.

**Figure 1:**
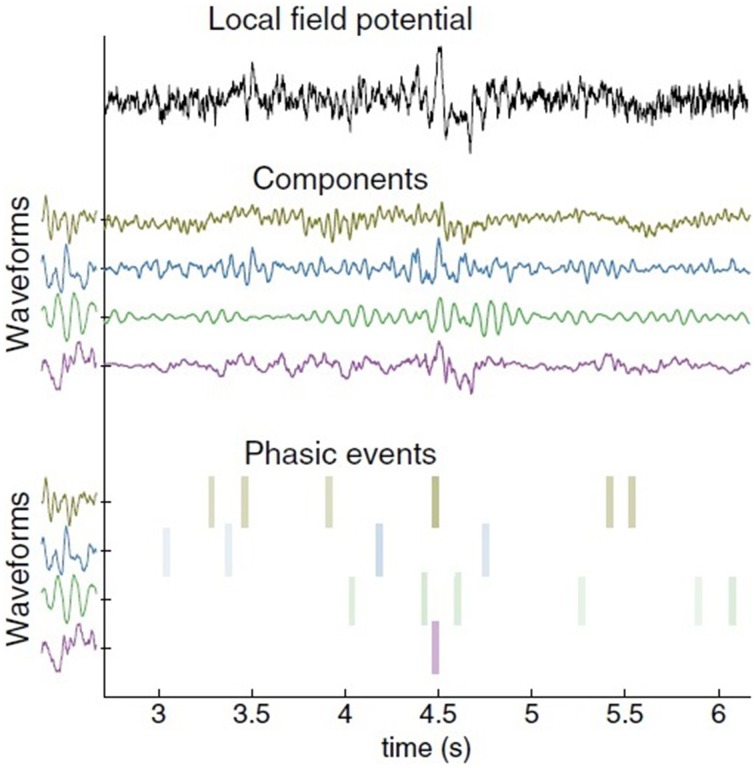
**Decomposition of a single local field potential (LFP) channel using the phasic event model**. Data collected by Brandi Marsh in Joseph Francis's laboratory at SUNY-Downstate. The original LFP signal is on top. In the middle are the component decompositions. The learned impulse response of the waveform is shown to the left of each component. The most significant amplitude atoms (timing, amplitude, and waveform index) appear at the bottom as colored bars. Color intensity corresponds to amplitude (from Principe and Brockmeier, 2015, p. 15).

Given these shortcomings, I would like to introduce the possibility of analysing a continuous signal as a linear combination of reoccurring waveforms. This is achieved by combining overcomplete representations with adaptive signal models. If the goal is to extract waveforms from a single continuous channel, then it follows that we should adopt a generative model which summates impulse responses, being a multiple input, single output (MISO) model. Principe and Brockmeier (2015, p. 15) term this a *phasic event model*. This proceeds in two steps: learning a set of waveforms occuring repeatedly throughout a signal, and estimating an atomic decomposition of a signal in terms of timing, amplitude, and waveform index (see Figure 1 for an example). The major advantages of this over other models is that the phasic event analysis learns the reoccurring waveform shape and allows the pinpointing of the amplitude and timing of phasic events. The model consequently captures the transitory nature of neural events.

Given the structure of the Basic Label model and the division of EEG patterns and local field potentials into rhythms (α, β etc.,) and phasic events (sharp waves, β and γ ripples etc.), I think an approximate correlational (in the sense of Embick and Poeppel, 2015) division between computations and representations can be established between, respectively, phasic events (carried out in and between the cell assemblies of particular regions) and rhythms (necessarily localized at such regions).

Although oscillations are likely not all that is needed to provide a solution to the problem of linguistic computation, they nevertheless appear to be a vital part of the answer. Aside from language-centered obstacles, comparative dynamic cognomics will also face the notable challenge of the variation in oscillation presence across species, with the reasons for much rhythmic variation still unknown. For now, the Basic Label model satisfies the cognome-dynome operational level, but we would ultimately want to satisfy the connectome and other lower levels. As a result, the next section will expand on the bare electrophysiological details outlined above leading to the broadening of multi-disciplinary concerns and perspectives.

## Biophysical directions

To adequately explore the neurochemical and biophysical details of the Basic Label model, it is useful to introduce a distinction between minimal and maximal degrees of explanation:

(1) a. Minimal degree of explanation (MinDE): The use of brain dynamics to explain why the cognome performs the operations it does, and not some other imaginable operations.
b. Maximal degree of explanation (MaxDE): The use of brain dynamics in addition to causally relatable accounts of neurochemistry and its underlying biophysics to explain why the cognome performs the operations it does, and not some other imaginable operations.

Note that MinDE has minimal requirements, whereas MaxDE has no stipulated limits, embracing the full range and plurality of the natural sciences. Neuroimaging studies, for instance, do not even reach the level of MinDE, whereas a purely rhythmic approach to the dynome of the kind found in Theofanopoulou and Boeckx (Forthcoming b) satisfies MinDE without reaching the neurochemical and biophysical precision of MaxDE. Kopell et al. (2014, p. 1319) stress that connectome-dynome linking hypotheses need to be supplemented with “the biological details that relate this connectivity more directly to function.” This is where I will attempt to depart from analyses which remain at the levels of the dynome and cognome (e.g., Sporns, 2013). For instance, Theofanopoulou and Boeckx (Forthcoming b) only refer in passing to basic interneuron classes, and their model lacks any serious neurobiological details. As Allen and Monyer (2015, p. 85) comment, “when considering interneurons, it would be important to investigate the role they play in the reactivation of cell ensembles occurring during sharp wave/ripples.”

Mechanistic ventures beyond the dynome are, I think, in the proper spirit of Turing's (other) thesis regarding morphogenesis, which was concerned not just with a description of an organism's forms (similar to the computational level of modern linguistics) but also with a proto-evo-devo theory of the cellular mechanisms which give rise to such forms (Turing, 1952, see also Maini, 2004). As Kopell et al. (2014, p. 1324) note, “an immersion in the physiology supporting temporal dynamics suggests mechanisms that would not be obvious if one were thinking abstractly about computation and rhythms”; a statement which carries urgent lessons for theoretical linguistics and neuroimaging.

Contrary to much of Koch's (1999) ambitious work, the following section will argue that the divide between biophysics and computation is in fact incommensurable, and that a different biolinguistic strategy will be required to resolve the granularity mismatch problem. This approach will use the Basic Label model alongside neurochemistry as tools to construct a neurobiologically feasible cognome, free of the technical baggage—though not the methodological naturalism (Chomsky, 2000; Collins, 2015)—of minimalist syntax and its lexico-centrism and “featuritis” (Boeckx, 2014a).

### Feeble currents and cognomic substrates

Though much interdisciplinary work remains to be carried out, dynamic cognomics has the potential to progress neurolinguistics beyond the situation described by Szathmáry in 1996: “Linguistics is at the stage at which genetics found itself immediately after Mendel. There are rules (of sentence production), but we do not yet know what mechanisms neural networks are responsible for each rule” (1996, p. 764). So far, I have only presented a model of how to embed the cognome within the dynome, but it is also vital to ground the dynome within the connectome and microlevel analyses, in turn addressing Szathmáry's concern.

It has been shown that neuronal populations can synchronously discharge due to an internal or external event, and additionally as a result of dynamic interactions between reciprocally coupled networks, which serve to “tag the responses of neurones that need to be related to one another,” as König (1994, p. 31) put it in his seminal assessment of neural oscillations. This synchronous activity further tends to be oscillatory in nature (Liu et al., 2010). Oscillations have also been linked to neurochemistry (Muthukumaraswamy et al., 2009), as discussed below. While oscillatory electrical activity in cell assemblies has been observed since the 1920s beginning with Berger's (1929) ground-breaking work, inspired by the Liverpool surgeon Caton's (1875) studies of the “feeble currents” generated by rabbit and monkey brains, its role in cognitive capacities has been intensively explored only since the new millennium (Jensen et al., 2002; Ossandón et al., 2011), largely down to theoretical, technological, and optogenetic advances. Updating Caton's imagery, McCormick et al. (2015, p. 133) summarize that brain rhythms are generated through “the interaction of stereotyped patterns of connectivity together with intrinsic membrane and synaptic properties.”

At the most common level of investigation, time-locked frequency analysis can decompose an EEG signal and identify changes in oscillations. But the widespread use of non-invasive and high-temporal resolution MEG, and recent advances in its source localization power (Wipf et al., 2010), have led to enhanced understanding of the spatiotemporal dynamics of oscillations and how they operate within neural networks. Recent work has begun to deliver an increasingly precise account of how, for instance, different classes of GABAergic interneurons in the hippocampus coordinate activity giving rise to network oscillations (Allen and Monyer, 2015), strengthening dynome-connectome correspondences. GABA_B_ receptors also perform time integration of cell assemblies (classically defined as a set of neurons exhibiting stronger within-group connectivity than with other connected neurons; Hebb, 1949) from the subsecond to second scale (Deisz and Prince, 1989), a vital function in computing conceptual and linguistic information representations.

Going beyond this level of analysis will require mapping rhythms to the numerous interneuron classes, which are defined based on cell body location, expression of marker proteins, axonal arborization, and other properties (Whittington and Traub, 2003; Klausberger et al., 2005; Somogyi and Klausberger, 2005). Korotkova et al. (2010) attempted to reach such a goal by showing how the removal of NMDA receptors in parvalbumin-expressing (PV) interneurons reduced the power of θ oscillations in the CA1 hippocampal region, while also reducing the γ-power modulation by θ oscillations. PV interneurons and somatostatin-expressing (SOM) interneurons preferentially synapse, respectively, onto the cell bodies and proximal dendrites of pyramidal cells and the distal dendrites of pyramidal cells (Royer et al., 2012). The silencing of PV interneurons, but not SOM interneurons, altered the θ phase precession in the brains of mice running on a treadmill belt in the experiments conducted by Royer and colleagues, suggesting that PV interneurons are highly fit to control the firing phase of principal neurons during θ oscillations, permitting the extension of a causal chain from cognome to dynome to a specific part of the connectome.

It should be noted, however, that PV and SOM expression is common to numerous hippocampal interneuron classes, and so further optogenetic work is needed in order to establish the role of individual interneuron classes in oscillation generation. Fruitful prospects for such work can be found in recent advances in juxtacellular recordings, permitting the monitoring of a single interneuron *in vivo*. To take a relevant case, Lapray et al. (2012) discovered that PV basket cells—providing inhibition to the pyramidal cell body and proximal dendrite—fire preferentially at the descending θ phase (findings reproduced by Varga et al., 2012), while ivy cells—providing inhibitory currents onto pyramidal cell dendrites—fire preferentially during ascension and at the trough. These studies reveal that during a single θ cycle the inhibitory power onto distinct pyramidal cell sectors varies systematically (see also Brandon et al., 2014).

Viewing cell assemblies as the fundamental unit of computation rather than single neurons can by now be justified in that assemblies can tolerate noise by not being redirected in their trajectory, unlike single or small clusters of neurons (which would also be effected by spike transmission failures), intensifying the justification for placing such assemblies at the center of the Basic Label model. Given the information chunking and feature merging roles attributed to γ cycles, Buzsáki suggests that episodes of γ oscillations, which contain strings of cell assemblies, “may be regarded as a neural word” (2010, p. 365); that is, a discrete unit of information. If induced γ is also responsible for constructing coherent conceptual objects by synchronizing neural discharges binding together distant brain regions, as proposed by Tallon-Baudry and Bertrand (1999), then oscillations may also be responsible for complex semantic phenomena like copredication, through which a single object or event can be conceptualized via simultaneously concatenated yet contradictory properties, e.g., *The newspaper I held this morning has gone bust* or *Lunch was delicious but took forever* (see Murphy, Forthcoming). Brain rhythms would consequently play a crucial role in constructing what Aristotle termed the “place of forms.”

Topics in electrophysiology should also direct the concerns of those investigating the brain dynamics of linguistic computation. Certain areas of recent research appear to be more commensurable with elementary computational operations than others. For instance, transfer of charges across membranes of all brain structures leads to a current giving rise to an extracellular field, which in turn influences the membranes. The transmembrane voltage (*V*_*m*_) is defined as the difference between the intracellular (*V*_*i*_) and extracellular voltage (*V*_*e*_) at a time *t* and location *x*: *V*_*m*_(*x*,*t*) = *V*_*i*_(*x*,*t*) – *V*_*e*_(*x*,*t*). A topic of contemporary debate is whether this endogenous field with its spatiotemporal *V*_*e*_-fluctuations changes neuronal functions through ephaptic coupling (see Jefferys, 1995 for an overview). This process amounts to a feedback mechanism through which the neural structures producing a given field are in turn affected by them, yielding a self-generated cyclic loop. In terms of range, ephaptic coupling influences structures ranging from synapses to discrete neurons to neural networks.

At the microscale, a linear relationship is seen between a chemical synaptic current *I*_*syn*_ and *V*_*m*_, with such current being able to be described as *I*_*syn*_(*t*) = *g*_*syn*_(*t*)(*V*_*m*_(*t*) – *E*_*rev*_), where *g*_*syn*_ is the synaptic conductance and *E*_*rev*_ is the reverse current. Following the above self-generated model, *V*_*e*_ changes alter synaptic currents. In addition, ephaptic coupling of *V*_*m*_ to electric fields influences spiking due to its effect on active cell conductances (Anastassiou et al., 2011). The explanatory force of ephaptic coupling becomes clearer with parallel plate whole-slice stimulation, which has shown that emergent properties of networks are more sensitive to electric fields than discrete neurons (Deans et al., 2007). As noted by Anastassiou and Koch (2015), the entrainment of spiking to field strengths as minimal as 0.5 mV/mm suggests that ephaptic entrainment to endogenous fields contributes to brain rhythms. Stronger ephaptic feedback also occurs after slower (< 8 Hz) waves such as θ and δ compared to faster γ waves, suggesting that the non-synaptic electrical signals seen in ephaptic coupling contribute to neural computation.

As with ephaptic coupling, I would additionally like to propose cross-frequency coupling (CFC) as a core component of computation, as discussed above. It has been suggested that this generic operation coordinates spatiotemporal neural dynamics (Canolty and Knight, 2010; Lisman and Jensen, 2013), resolving a long-standing problem over how neural activity is synchronized. With larger neuronal populations oscillating at lower frequencies and smaller populations doing so at higher frequencies, CFC would enable their synchronization. In particular, it has been shown that via “phase-amplitude” CFC the phase of the lower frequency modulates the amplitude of the higher frequency component, a process claimed to be involved in information transfer for faculties such as memory (Tort et al., 2009, though see Aru et al., 2015 for current limitations of phase-amplitude modeling).

But while much is known about the biophysical substrates of individual frequency components, the cellular mechanisms behind frequency *interactions*—the origin of linguistic computation in the Basic Label model—remain opaque. Initial research leading to such an account has already been mentioned: Recall Korotkova et al. (2010) and their findings regarding hippocampal θ•γ coupling and its reliance on NMDA receptor-mediated PV interneuron excitation (see also Bi and Poo, 1998; Tort et al., 2008). Using laminar electrodes to measure activity in monkey primate visual cortex, Spaak et al. (2012) found that α phase in infragranular layers modulates γ amplitude in supergranular layers (see also Friston, 2008); similar to how thalamic nuclei oscillating at the α band synchronize distant cortical regions oscillating at higher frequencies. As Aru et al. (2015) note, the most elegant theory to account for these findings is that periodic membrane potential fluctuations generate low frequency oscillations which subsequently gate the incidence of higher frequency activity in a phase-specific fashion. From a functional perspective, the above nested γ cycles could act as multiplexing mechanisms (Buzsáki, 2006, p. 356) for sustaining working memory representations by sending multiple representations as a single complex message to be recovered and “unpacked” downstream (see Hyafil et al., 2015 for empirical support, and Baddeley et al., 2014 for a review of working memory mechanisms); precisely as is seen in labeling and phasal transfer.

At a more general level, the cognome must operate within certain fundamental constraints on neuronal dynamics, such as the free-energy principle (following seminal insights from Friston, 2010) through which the homeostatic brain minimizes the dispersion (entropy) of interoceptic and exteroceptic states. If entropy is the average of “surprise” over time, then the brain will choose appropriate sensations to minimize surprise, and in so doing “the brain is implicitly maximizing the evidence for its own existence” (Bastos et al., 2012, p. 702); a notion not too far removed from Vaas's assessment that the brain is “a self-referential, closed system, a functional reality emulator that constructs the world, rather than reconstruct it” (Vaas, 2001, p. 88). This form of “predictive coding” conforms to the free-energy principle and the image of the brain as a *constructive organ*, assembling and inferring linguistic representations. Studies of chaotic itinerancy (Tsuda, 2013, 2015), many-body physics and thermodynamics (Vitiello, 2015) may also prove indispensable in describing the high-dimensional state space of cortical activity implicated in computation (see the essays collected in Ohira and Uzawa, 2015 for discussion).

**Figure 2:**
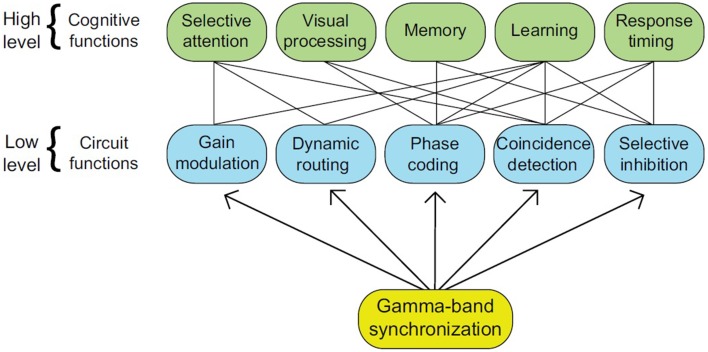
**The numerous roles attributed to gamma-band synchronization (GBS) are represented by the higher tier, while their implementation in neural circuits is represented by the lower tier (from Bosman et al., 2014, p. 1983)**.

An emerging consensus regarding the validity of the communication-through-coherence (CTC) hypothesis lends further impetus to the claim that rhythms bring about linguistic computation (Bastos et al., 2015). CTC claims that rhythmic synchronization, especially in the β and γ bands, modulates the efficacy of anatomical connections, and that oscillations are necessary for long-distance assembly formation (König et al., 1995; Fries et al., 2008). CTC can be complemented with recent developments in the understanding of the functional role brain rhythms play, with assembly formation being the core operation at the connectome level necessary to establish the kinds of cross-modular representational structures seen in natural language (Lopes-dos-Santos et al., 2011). γ band activity, for instance, has been associated with numerous cognitive functions such as memory and selective attention (see Figure 2 for examples of connectome-cognition links). With γ bands arising from an interplay of inhibition (produced by GABAergic neurons) and excitation (produced by glutamergic neurons), Bosman et al. (2014) propose that these bands have their origin in basic functional motifs conferring an advantage for low-level system processing and multiple cognitive functions (see also Bartos et al., 2007; Buzsáki and Wang, 2012).

The broad functionality of γ makes it an ideal candidate, along with the thalamus (discussed below), for being the conductor of language's cross-modularity. The role of GBS in visual feature integration (Bosman et al., 2009), for instance, makes it a prime candidate for carrying out the forms of conceptual assimilation seen in any number of semantic phenomena. If linguistic computations are in fact responsible for this cross-modularity, then language can perhaps be more closely aligned to dominant descriptions of consciousness and working memory (Dehaene et al., 2014), even if we are forced to remain “virtually mute” (Chomsky, 1998, p. 440) about the nature of experiential content (Strawson, 2008, 2010).

**Figure 3:**
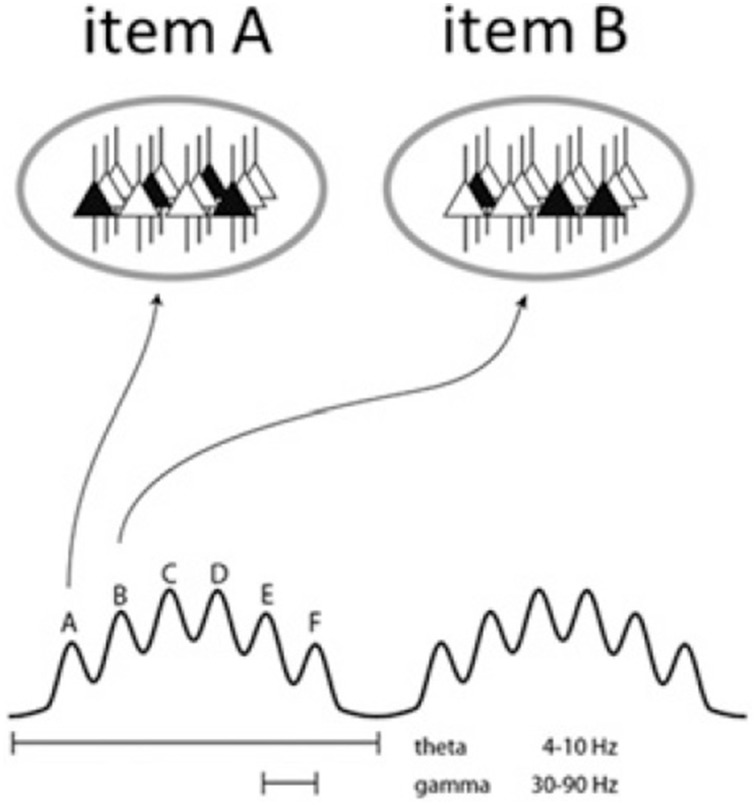
**Schematic of the theta-gamma neural code**. The ovals represent states of the same network during two gamma cycles. Active cells are in black and represent the cell assemblies which code for a particular item, i.e., memory units or, under the Basic Label model, conceptual representations and lexical features. Different assemblies are active in different cycles (from Lisman and Jensen, 2013, p. 1003).

In addition, GBS has been shown to support certain low-level functions in the hippocampus which may be vital to particular cognitive functions attributed to this region, such as memory encoding and retrieval (Bosman et al., 2014). As mentioned, the hippocampus is the site of γ•θ coupling in that multiple γ waves are typically embedded within a single θ cycle (Bragin et al., 1995). Along with the standard phase locking operation through which higher waves occur at stable phases in cycles of lower waves (Belluscio et al., 2012), this allows spike coordination and may consequently be partly responsible for low-level dynome operations like phase coding (see Figure 3). As Lisman and Jensen (2013) review, the dual γ and θ oscillations form a code for representing multiple items in an ordered way. Since each θ cycle contains four to eight nested γ cycles, different forms of spatial information (such as a series of events from short-term memory, constituting an “episode”) can be represented and sequentially coordinated within a given cycle. This may in turn constrain the number of lexical items or features able to be transferred in a given phase. Through the coding scheme discussed by Lisman and Jensen, the cell assembly that fires during a given γ cycle forms a topographic pattern representing a particular item from memory. If this oscillatory mechanism is also responsible for syntactic computation, this would lend weight to the strong connection drawn in Murphy (2015b) between syntactic phases and episodic memory. The number of γ cycles able to be embedded within a θ cycle may also be the reason why working memory is limited to its classic constraint of 7 ± 2 (Kamiñski et al., 2011). Roux and Uhlhaas (2014) make the related claim that oscillatory activity assures the maintenance of working memory information. This explanation is of precisely the kind of granularity linguists should seek to capture syntactic operations like labeling, which involves storing conceptual roots in memory. In brief, and returning to issues outlined above, if intrinsic coupling across cortical oscillations is responsible for the hierarchical combination of computations at the syllabic and phonemic levels, “restoring the natural arrangement of phonemes within syllables” (Hyafil et al., 2015), then this leads to the possibility that hierarchical syntactic computations result from similar mechanisms.

These operations are all conserved from early in mammalian evolution, with the above interplay between excitation and inhibition being found in crustaceans (Nusbaum and Beenhakker, 2002) and major phyla dating back 350 million years (Katz and Harris-Warrick, 1999). Bosman et al. (2014) draw on such considerations in claiming that the evolutionary acquisition of this excitation-inhibition interplay led to the selection of these γ waves as a principal element of computation. If this GBS mechanism was a “direct, inevitable consequence of early circuitry organization” (Bosman et al., 2014, p. 1994), then it may be that it is an exaptation (being co-opted) in that it was later afforded a functional role in systems of memory and learning (see also Gould and Vrba, 1982). Further, top-down neocortical processes implicated in particular higher cognitive faculties like working memory (Buschman and Miller, 2007) and free-choice reach (Pesaran et al., 2008) also appear to be carried by interareal synchrony in the β rhythm (Bressler and Richter, 2015), increasing the electrophysiological validity of the functional roles attributed to this wave above.

### Cognomic constraints and their neurobiological realizability

In the same way that γ oscillations “arise simultaneously and inevitably with inhibitory-excitatory interplay, and are neither an epiphenomenon nor a separate cause of the functionality beyond the underlying circuits” (Bosman et al., 2014, p. 1995), I would like to suggest that “linguistic” “computations” (which, as discussed, are neither purely linguistic nor thoroughly computational) are to be seen as identical to the operations of the connectome, which can be described in electrophysiological terms at the dynome level and in still more abstract terms at the cognome level, in a similar way that *heat* and *energy* can be reduced to thermodynamics. While I hope to have shown that distinct oscillatory phases segregate discrete units of information (visual, olfactory, semantic, etc.,), there remains the possibility that they also serve computations spanning multiple oscillatory cycles. Oscillatory phases may be the means through which different lexical features (e.g., φ, tense) are processed or time-locked with other features, leading to agreement relations, the resolution of filler-gap dependencies, feature inheritance/copying, and other familiar syntactic operations. Multiple β or θ cycles could, for instance, employ dynomic operations like “cycle skipping” (Brandon et al., 2013) to control which cell assemblies are activated upon subsequent cycles to trigger different aspects of lexical and conceptual representations.

These remarks cover some basic computations, but what of their *constraints*? Consider Wurmbrand's (2014) Merge Condition, stated below:

(2) *Merge Condition*:
Merge α and β if α can value a feature of β.

This condition ensures that set-formation via concatenation is licensed only under Agree, requiring also feature valuation. Leaving aside further details and the possibility that Merge applies freely, the scientist concerned with establishing linking hypotheses between linguistics and neuroscience is faced here with a number of challenges but also some surprising possibilities. For example, the cell assemblies implicated via cycle skipping in the features of α and β may undergo phase-locking, leading to oscillatory synchronization of two discrete units of information. When this occurs, feature valuation takes place and the derivation can converge. If this process is barred in virtue of rhythmic coupling restrictions and the limits of assembly synchronization, feature valuation, and hence concatenation, does not take place. If the distribution of unvalued features, [*u*F], also contributes to the demarcation of phases (Narita, 2014a), then the dynamics of feature valuation would likely align closely with the present Basic Label account of Transfer, since valuation, Agree and other copy-forming operations such as Internal Merge apply as a fundamental part of Transfer. Notice that this model at once implies specific neurobiological limitations, in that the hypothetical coupling responsible for feature valuation should occur after the cross-cortical {α(γ)} embedding proposed to be the substrate of set-formation. This leads to clear, causally-addressable empirical predictions, to be investigated in future research.

As a secondary concern, I will assume that feature valuation (along with feature inheritance and Agree) are both cases of a more generalized Search operation, which forms relations between identical feature complexes (Ohta et al., 2013; Kato et al., 2014). Kato et al. (2014) even go as far as claiming that Search is in turn just an instance of Merge, and that the human language faculty may reduce to pure Merge. The Basic Label model and Kato et al. consequently yield different predictions about the dynome. From here, the matter is purely empirical, but these subtleties in distinct cognome-dynome hypotheses are yet to be investigated and are potentially of substantial interest to dynamic cognomics.

We are now in a position to outline a concrete research program. The first phase of dynamic cognomics will involve the above ongoing research into translating or reconstructing the *operations* of syntax into oscillation terms. The second phase should center on translating the *constraints* of syntax, such as those concerning agreement, movement, and anti-locality. For instance, Richard's (2010) Distinctness Condition, prohibiting the presence of multiple lexical units of the same label within a single phase complement, may be the consequence of how many distinct rhythms it is possible to couple in specific actions (Boeckx, 2013). These ^*^XX-like structures (e.g., structures containing multiple phase-internal nouns such as ^*^John Mary ate apples) may be ungrammatical because of the oscillatory patterns local language regions can sustain. These constraints may form the backdrop of what Narita (2014a, p. 26) identifies as a core aspect of minimal computation, the “Minimal Workspace” through which the construction and transferring of syntactic structures takes place. To put it more concretely, language-external systems (interfaces) may only be able to sustain a single rhythm from the γ and β bands due to the small size of localized regions, and would hence be incapable of interpreting multiple category-identical elements in a single cycle. The phase/non-phase rhythm of syntactic computation would thus arise from the limits of oscillatory sustainability, and the connection between syntactic phases and oscillatory phases becomes more than purely orthographic: [C [T *v*[V D/*n* [N]]]] emerges from [β [γ β[γ β [γ]]]] given the labeling role attributed to β above, which in turn explains ^*^XX violations. Narita's (2014b) ^*^{*t*,*t*} constraint, which prohibits the transfer of syntactic objects whose two members are both traces/copies of movement, also strikes me as amenable to a similar, if not identical explanation. Objects of the {*t*,*t*} kind cannot be labeled, as in (3), and are hence illicit (Moro, 2006, p. 15):

(3) ^*^[which picture of the wall]_*i*_ do you think that [the cause of the riot]_*j*_ was {*t*_*i*_,*t*_*j*_}?

What is needed is consequently a re-conceptualization of language as not only a system of thought, planning and interpretation, but also a system of oscillatory and electrophysiological information synchronization. The computational constraints explored by Wurmbrand and others can direct inquiry into the possibilities of dynomic operations, although this process may require further elaboration of the nature of the role of oscillations in cognition.

### Globularity and cortico-centrism

**Figure 4:**
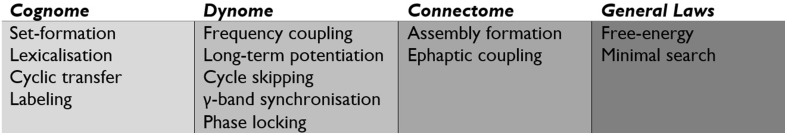
**The central operations implicated by the Basic Label model of the cognome, dynome, and connectome, along with more general laws**.

Recent developments in systems neuroscience have identified large scale distributed brain networks, typically explored through fMRI and MEG (Brookes et al., 2012). Data from fMRI suggests that the implication of a functionally specific set of neurons in any given computation is assisted by a backdrop of large-scale neural assembly inter-communication. These networks are composed of sub-networks with correlating and anti-correlating patterns, leading to a situation in which a single large-scale network may operate through overlapping but distinct neural sub-networks. Figure 4 highlights the major operations at the level of the cognome, dynome, and connectome, along with general laws influencing such operations.

As the cognome-dynome-connectome linking hypotheses expand, it is important not to ignore the fundamental role of the genome. Consider briefly the genes *RUNX2*, the *DLX* suite and the *BMP* family, involved in skull and brain development (Perdomo-Sabotal et al., 2014). In a series of ongoing research, Boeckx and Benítez-Burraco (2014a,b, Benítez-Burraco and Boeckx, 2015) hypothesize that a modification in this gene network gave rise to a more “globular” head shape (relative to Neanderthals/Denisovans; Bruner, 2004; Gunz et al., 2012; Theofanopoulou, 2015)—approaching a level of sphericity unseen in our closest ancestors—and the consequent re-wiring of cortical and sub-cortical structures, permitting the construction of the forms of cross-modular representations well established in psychological, philosophical, and semantic theories of concepts (Spelke, 2010; Pietroski, Forthcoming). Globularity may also have contributed, as some have suggested, to an increase in wiring efficiency across the brain (Chklovskii et al., 2002). It is of outstanding interest for biolinguistics and dynamic cognomics that functional links of this kind are beginning to be drawn between genes and their cellular consequences for the human cognitive phenotype.

An evaluation of these observations can also be made alongside a consideration of what Piattelli-Palmarini and Uriagereka (2008) see as the optimizing role language has in building syntactic and phonological structures, which proceeds via minimal search and related principles of computational efficiency (Larson, 2015). This minimalist perspective leads to a separation of optimality from language's proposed “function” of mapping structures to the interfaces, since similar optimizing principles are found elsewhere in the natural world, leading Piattelli-Palmarini and Uriagereka (2008, p. 209) to “suspect that the process behind the abstract form follow[s] from physico-chemical invariants.” But lacking a theory of brain dynamics, the authors are unable to ground these general proposals within any neurobiological framework. I suggest that the microcellular level and the dynome, operating within some general physical laws of neural organization such as free-energy, can provide a potential substrate of such “physico-chemical invariants.” The only human-unique aspect of the model pursued here, then, is the *context* in which the conserved and universal rhythms discussed above perform their operations of coupling and decoupling; namely, a globular brain case, which would have led to a decrease in the types of “spatial inequalities” (Salami et al., 2003) between cortical and subcortical regions which would prohibit long-distance coupling. This would imply that the numerous centuries-long approaches to human-uniqueness, ranging from philosophy to medicine, have approached the matter from the wrong perspective. Instead of asking what it is about humans which allows us to form complex systems of symbolic interpretation, we should instead ask what it is about other animals which *prohibits* them from doing so.

Globularity may also have led to the expansion of the neo-cortex and the pulvinar, spurred on by the reduction of the large Neanderthal visual system (Pearce et al., 2013). As Benítez-Burraco and Boeckx (2015) point out, cross-modular concepts likely employ thalamic nuclei such as the pulvinar and the medio-dorsal nucleus, not least because of the thalamus's role in modulating fronto-parietal activity, regulating cortical oscillations (Saalmann et al., 2012) and enhancing the rhythmic range of different frequency bands (Singer, 2013). Controlling rhythmic behavior is also a function attributed to *RUNX2* (Reale et al., 2013, see also van der Lely and Pinker, 2014 for genetic discussion relating to phonological computations). A literature review leads Theofanopoulou and Boeckx (Forthcoming a,b) to claim that the thalamus is the brain region which tunes the oscillations of other subcortical structures (see also Boeckx, 2014b). The importance of the thalamus for higher cognition was also speculated in work by Campion and Elliot-Smith (1934), rejecting the dominant cortico-centrism and suggesting that cortico-thalamic impulse circulation was responsible for “thought.”

Relatedly, due to the few protein differences between humans and chimpanzees, the individuating computational factors may be attributed to cis- and trans-regulatory genes (Somel et al., 2013). Hominid-unique features which may have led to the higher mental faculties of humans include novel neuronal cell types and the duplication of developmental proteins such as SRGAP2, leading to unique dendritic spine density and form (Geschwind and Rakic, 2013). Synaptic and dendritic maturation also occurs in humans for a considerably longer time than in non-humans (Bianchi et al., 2013). If we also consider the conclusions of Harris's review of cortical computation in mammals and birds, that the “human cortex appears to contain the same cell types, and their patterns of wiring and gene expression appear basically similar to well-studied model systems” (2015, p. 3184), the importance of subcortical investigations into linguistic computation becomes even clearer. While subcortical structures have often been derided as the “reptilian brain,” responsible for only primitive drives, far removed from the neocortex's higher echelons of thought, the perspective of dynamic cognomics re-situates subcortical regions like the thalamus and the basal ganglia into the core areas responsible for linguistic phrase structure building (see also Johnson and Knight, 2015 for evidence that the thalamus plays a key role in neocortical oscillations involved in memory processes).

Summarizing these findings, it appears that the developed interneurons and dendritic spinal strength proposed by Geschwind and Rakic (2013) fortified long-distance assembly connections and, in turn, the mechanisms of ephaptic coupling, CFC and other neuronal processes (operating within the confines of the CTC hypothesis) necessary for the rhythmic interactions claimed above to be the source of computations like labeling and cyclic transfer. The targeting of the perisomatic region of pyramidal neurons by inhibitory interneurons in particular leads to the formation of γ rhythms and their concomitant properties of conceptual assimilation. Though many intervening neurochemical processes need to be accounted for and explained, it seems that such processes, along with novel *V*_*e*_-fluctuations, are the reason why we find the cyclic short-term memory storage capacities seen in labeling. Updating Darwin's claim of “He who understands baboon would do more toward metaphysics than Locke,” we can conclude that he who understands brain rhythms would do more toward biolinguistics than Lenneberg.

#### Conflict of interest statement

The author declares that the research was conducted in the absence of any commercial or financial relationships that could be construed as a potential conflict of interest.
